# Parsing cell death in arrhythmogenic cardiomyopathy: PANoptosis

**DOI:** 10.20517/jca.2022.45

**Published:** 2023-01-12

**Authors:** Calum A. MacRae

**Affiliations:** Cardiovascular Medicine, Network Medicine and Genetics Divisions, Brigham and Women’s Hospital, Harvard Medical School, Boston, MA 02115, USA.

## Cell death as a way of life

There are many ways for a cell to die and each of the modes of cell death has been subject to intense evolutionary selection pressure^[1]^. Discrete survival pathways and related programmed cell death (PCD) pathways have emerged in single cells, across communities of cells, and in all multicellular organisms. Many of the factors which trigger programmed cell death act through perturbations in homeostatic parameters such as osmotic pressure, mechanical force, temperature, oxygen tension, pH, transmembrane potential, DNA damage or metabolic substrate availability. Although they use distinctive sensor systems and signaling pathways, microbial or other injuries and their responses can often be understood using these same rubrics and the survival of whole organs and organisms can be framed through community behaviors that typically integrate heterogeneous responses to single insults across different cells or cell types^[2]^.

In cell communities ranging from biofilms to human hearts, PCD pathways have also evolved critical signaling roles, driving downstream responses at the whole organism level that mitigate the effects from individual upstream insults^[2]^. With microbial infections, these pathways constitute stereotypic innate or acquired immune responses, but with less well-defined insults, the outcomes may be less readily characterized as an integrated response profile and typically are classified more broadly in generic pathological terms such as inflammation, atrophy, hypertrophy, fibrosis and dystrophy^[2]^.

Programmed cell death has been characterized on the basis of specific protein complexes which act to transduce particular classes of danger signals via a series of different effector complexes which result in discrete patterns of cellular demise, labeled as apoptosis, pyroptosis or necroptosis^[2]^. The effector complexes are triggered to assemble by pattern recognition receptors of various types that sense exogenous pathogens or DNA damage, as well as a range of other cellular danger signals. The core features of apoptosis include a loss of mitochondrial membrane integrity with cytochrome c efflux into the cytoplasm and activation of a caspase cascade which can also be independently triggered by engagement of a growing list of plasma membrane death receptors. These two input pathways can also interact synergistically. Apoptosis has classically been thought of as “non-lytic” as it does not involve plasma membrane rupture or cytokine release with the associated inflammatory responses these events would drive. However, apoptosis itself generates internal danger signals which can trigger other forms of PCD and the caspases at the heart of apoptosis can also participate in these other routes to cell death. Necroptosis is a lytic form of PCD induced by a series of membrane receptors, which in the absence of caspase-8 activity lead to the formation of a distinctive cytosolic complex, generation of a phosphorylated mixed lineage kinase domain-like protein oligomer, plasma membrane disruption and cell lysis^[2]^. Finally, pyroptosis acts through inflammasomes, each of which has a set of specific pattern recognition receptors and effector molecules, but which share the recruitment of proteins that cooperate to cleave pro-forms of cytokines and disrupt the plasma membrane to kill the cell and release a series of proinflammatory molecules including IL1β, IL18 and cellular distress signals^[2]^. Not unexpectedly, given the very particular contexts in which each of these forms of PCD have been described, there is clear evidence that both pyroptosis and necroptosis can activate apoptotic pathways through broader caspase recruitment or lysis of intracellular membranes, among other cross-talk mechanisms, leading to apoptosis. Lytic PCD pathways have also been shown to exhibit substantial cross-talk, not least through the central membrane events which include effects on potassium currents in addition to the pore forming capabilities of the necrosome and pyrosome^[2]^. Emerging forms of PCD such as ferroptosis, parthanatos, oxeioptosis and NETosis have yet to be fully integrated into broader frameworks, but the generalizable insight that cell death triggers is more likely an integrative rheostat rather than a set of rigid switches remains valid.

The extensive cross-talk at different levels between each of the PCD pathways and the distinctive outcomes observed with particular insults suggest quite granular integrative events which include the cumulative effects of the insult itself, baseline cell state, and the effects on each of the PCD pathways across a wide dynamic range. This threshold view of PCD has been bolstered recently by the description of the phenomenon of PANoptosis, which combines components from each of the other PCD pathways into a single multiprotein complex characterized by the presence of the Z-DNA binding protein 1 (ZBP1) as well as components of the inflammasome, necrosome and apoptotic caspases^[2,3]^. The precise composition of this PANoptosome appears to depend on the specific stimuli and the particular cellular context, further reinforcing the concept of a much more precisely tailored set of graded cell death decisions than the limited number of PCD switches in the traditional canon^[2]^.

The discordance between traditional views of PCD and this broader framework reflects not only the wide repertoire of potential death triggers, only a small fraction of which have been rigorously studied, but also our patchy insight into some of the determinant signals such as complex lipid contributions (both within cellular membranes but also in biological condensates), unmeasured physical or chemical mediators (e.g., mechanical forces or self-organizing molecular behaviors), and the partitioning of such signals within discrete intracellular compartments. Some of these same unknowns are hinted at by the genetics of PCD in development, where coordinated cell death is critical for physiologic patterning of the organism and is regulated by the canonical pathways including Wnt, Notch and BMP which also appear to have salutary effects on some forms of disease-related PCD^[4,5]^. Finally, perhaps one of the most important determinants of these complex biological outcomes is timing, with different forms of PCD acting downstream of the same stimulus at different times. In this setting, chronic disease models may offer distinctive insights into the interactions among different cell death modalities.

## Cardiomyopathy and PANoptosis

The arrhythmogenic cardiomyopathies (ACMs) are a group of cardiomyopathies distinguished by a propensity to arrhythmias with distinctive mechanisms typically observed in the regions of the heart derived from the second heart field^[6]^. The original syndrome was identified through the structural involvement of the right ventricle (RV) with fibro-fatty “infiltration” or transdifferentiation and reentrant RV arrhythmias associated with epicardially based scars, so-called arrhythmogenic RV cardiomyopathy (ARVC). Subsequent studies have identified the common involvement of genes encoding junctional proteins contributing to desmosomes, but also observed similar phenotypes in cardiomyopathies caused by mutations in the genes encoding LMNA, TMEM43 and other proteins less clearly participating in the desmosome^[7]^. There is also well-described overlap between the phenotypes observed in ARVC and those in LV cardiomyopathies associated with the same genes, or the phenotypes observed in conditions that on first inspection appear distinct, including RV outflow tract (RVOT) tachycardias and other proarrhythmic syndromes with less specific structural findings. High-resolution phenotyping, such as intracardiac electrophysiology, does allow the discrimination between the scar-associated arrhythmia thought typical of ARVC and the cAMP-mediated triggered events thought to be benign reflections of pure RVOT tachycardias. However, these tools have not been deployed rigorously in the clinic and there is no doubt that both arrhythmia mechanisms can coexist^[8]^. One related insight is the potential shared mechanisms between scar-related and developmentally-defined transition zones with respect to sustained electrical reentry.

Desmoplakin (DSP) is a representative of the plakin family, a set of obligate desmosomal proteins which link the desmosomal plaque to intermediate filament cytoskeletal network and also, in ways less well understood, to the microtubular network^[7]^. In addition to these core structural roles, anchoring cells to each other and their internal cytoskeletal networks, there is also a wealth of evidence that DSP has important functions in cell signaling and regulating the dynamics of desmosome turnover. These features are of note in the setting of emerging roles for conformation-induced conformational changes in desmosome maturation and in the assembly and turnover of desmosomal cadherins. Cadherins themselves are an integral contributor not only to transmembrane junctional complexes but also to intracellular membrane trafficking and targeting to specific subcellular compartments. It is clear that there are multiple potential mechanisms by which the repertoire of desmosomal proteins might act in the reception, transduction or execution of PCD signals.

In this issue of the Journal, Olcum and colleagues outline detailed analyses of a murine model of DSP cardiomyopathy, which is characterized, as is the human disorder, by prominent cell death with subsequent inflammation and fibrosis^[6]^. Using an inducible cardiomyocyte-specific biallelic deletion of DSP, the authors were not only able to recapitulate the clinical DSP cardiomyopathy phenotype, but also to unravel the temporal trajectories of different components of the underlying molecular and cellular pathogenesis. The cardiac-restricted DSP deletion was induced at postnatal day 14 and the mice were then studied 4 weeks later before they began to die at a median of ~2 months of age.

The authors were able to exploit the inducible genetic deletion and the abbreviated pathogenetic timeline to characterize the molecular response to cardiac-restricted DSP deficiency in detail. They elegantly document the contractile defects, arrhythmias and consistent epicardially-based myocardial fibrosis, which replicate findings in human DSP cardiomyopathy. Using bulk RNA-seq, they demonstrate the anticipated activation of EMT, inflammation and apoptosis, as well as the suppression of oxidative phosphorylation and fatty acid oxidation that define the discrete cell states previously observed in defined forms of ACM. Importantly, they delve more deeply into the activation of PCD, confirming at a transcriptional and translational level the activation of death-receptor and mitochondrially mediated apoptosis. Taking an unbiased approach, they go on to define concomitant activation of necroptosis and pyroptosis in the DSP null hearts at transcript and then protein levels. Finally, given the evidence that multiple PCD pathways are activated, the authors assess the expression of the transcript encoding the proposed master regulator of this so-called PANoptosis, Z-DNA binding protein 1 (ZBP1), and find it substantially upregulated in DSP null hearts^[3,6]^.

The study by Olcum *et al*. acknowledges some key limitations, particularly the representativeness of the conditional homozygous DSP deletion and the background effects of the MerCreMer fusion protein^[6]^. Nevertheless, the questions raised by this interesting study are manifold and the authors have nicely established a rigorous baseline for a deep exploration of several of these areas using their model. For example, what are the relative contributions to DSP cardiomyopathy from each of the PCD pathways and how are these discrete mechanisms integrated over time? The inducible nature of the DSP disease model will enable these elements to be dissected over a wide temporal range. Second, what are the regional contributions to the final outcome in individual cells or across the whole organ? The deployment of single cell and spatially defined functional genomics will allow the authors to explore developmental origin, mechanical forces, specific molecular signals and other factors that define the gradients of cellular death and downstream effects such as fibrosis that are apparent across the myocardial thickness from epicardium inward. Understanding the transition boundaries and their determinants will offer fundamental insights into the cell characteristics that dictate particular PCD outcomes in the face of seemingly identical danger signals. Another question that the inducible DSP model is well placed to address is the extent to which intercellular physical interactions may define the events leading to PCD activation. The desmosomal cardiomyopathies as a class have been shown to be suppressible by GSK3β modulators, suggesting that at least some components of cell death can be understood in terms of canonical Wnt signaling and can be prevented or diminished by inhibition or tuning of this pathway^[4,5,7,9]^. Understanding these signals at a quantitative level in a disease setting may offer an inroad into the use of Wnt modulators for benefit in chronic disease.

## Striving for generalizable outcomes

Ultimately, it is difficult to escape the feeling that at our current level of mechanistic insight, almost everything in biology is connected to everything else. The panoply of trajectories to PCD reflects the diversity of insults and the diversity of competing survival pathways. There is a strong argument for a systems approach to exploring disease biology defined by relevant models studied across all of the potential outputs such as cell death, inflammation, fibrosis, contractility and arrhythmia, so we can define inputs and outputs without the overlay of timing or preconceived linear pathways. Olcum *et al.* highlight the advantages of looking at “all” of the mechanisms in a precise model and define the potential for a more rigorous mechanistic understanding of ACM across the entire trajectory of the disease^[6]^.

## Data Availability

Not applicable.
